# Publisher Correction: BIN1 knockdown rescues systolic dysfunction in aging male mouse hearts

**DOI:** 10.1038/s41467-024-48944-4

**Published:** 2024-05-23

**Authors:** Maartje Westhoff, Silvia G. del Villar, Taylor L. Voelker, Phung N. Thai, Heather C. Spooner, Alexandre D. Costa, Padmini Sirish, Nipavan Chiamvimonvat, Eamonn J. Dickson, Rose E. Dixon

**Affiliations:** 1 Department of Physiology and Membrane Biology, University of California Davis, Davis, CA USA; 2 Division of Cardiovascular Medicine, Department of Internal Medicine, University of California, Davis, Davis, CA USA; 3 Department of Veterans Affairs, Northern California Health Care System, Mather, CA USA; 4 Department of Pharmacology, University of California Davis, Davis, CA USA

**Keywords:** Ageing, Cardiovascular biology, Cardiovascular biology

Correction to: *Nature Communications* 10.1038/s41467-024-47847-8, published online 25 April 2024

The peer review file was originally published with confidential data intended solely for the Reviewers and Editors; this material has now been redacted and the peer review file has now been replaced with the correct redacted version.

### Supplementary information

Updated Peer Review File

