# Supplementary material for: BIN1 knockdown rescues systolic dysfunction in aging male mouse hearts
Source: Nat Commun. 2024 Apr 25;15:3528. doi: 10.1038/s41467-024-47847-8 (PMC11045846; doi:10.1038/s41467-024-47847-8)

# Supplementary Information

## BIN1 knockdown rescues systolic dysfunction in aging male mouse hearts

Maartje Westhoff Ph.D.[1]*, Silvia G. del Villar Ph.D.[1]*, Taylor L. Voelker Ph.D.[1], Phung N. Thai Ph.D.[2], Heather C. Spooner[1], Alexandre D. Costa Ph.D.[1], Padmini Sirish Ph.D.[2], Nipavan Chiamvimonvat M.D.[2,3,4], Eamonn J. Dickson Ph.D.[1], and Rose E. Dixon Ph.D.[1]

** These authors contributed equally to this work.*

Information within this file:

**Supplementary Figure 1.** Comparison between young C57Bl/6 mice sourced from the Jackson Laboratory (JAX) and the National Institute on Aging (NIA).

**Supplementary Figure 2.** Time course of $I_{Ca}$ response to ISO in young and old myocytes.

**Supplementary Figure 3.** Time course of calcium transient response to ISO in young and old myocytes.

**Supplementary Figure 4**. Time course of ISO-stimulated $Ca_V1.2$ and RyR2 clustering in young myocytes.

**Supplementary Figure 5.** Transferrin receptor recycling is slowed in old myocytes.

**Supplementary Figure 6.** BIN1 isoforms present in the heart.

**Supplementary Figure 7.** Further characterization of BIN1 knockdown in old mice.

**Supplementary Figure 8.** Youthful t-tubule network organization is recovered in old myocytes upon BIN1 knockdown.

**Supplementary Figure 9.** EEA1 positive early endosomes are not altered with BIN1 knockdown.

**Supplementary Figure 10.** Additional data from conscious mice using M-mode echocardiography.

**Supplementary Figure 11.** Left ventricular dimensions of conscious young and old mice from M-mode echocardiography.

**Supplementary Figure 12**. Additional data from unconscious mice using M-mode echocardiography.

**Supplementary Figure 13.** Analysis of systolic function in unconscious mice with and without ISO.

**Supplementary Table 1.** ISO-stimulated changes in peak $I_{Ca}$ and voltage dependence of $G/G_{max}$ of young and old ventricular cardiomyocytes.

**Uncropped Western Blots**

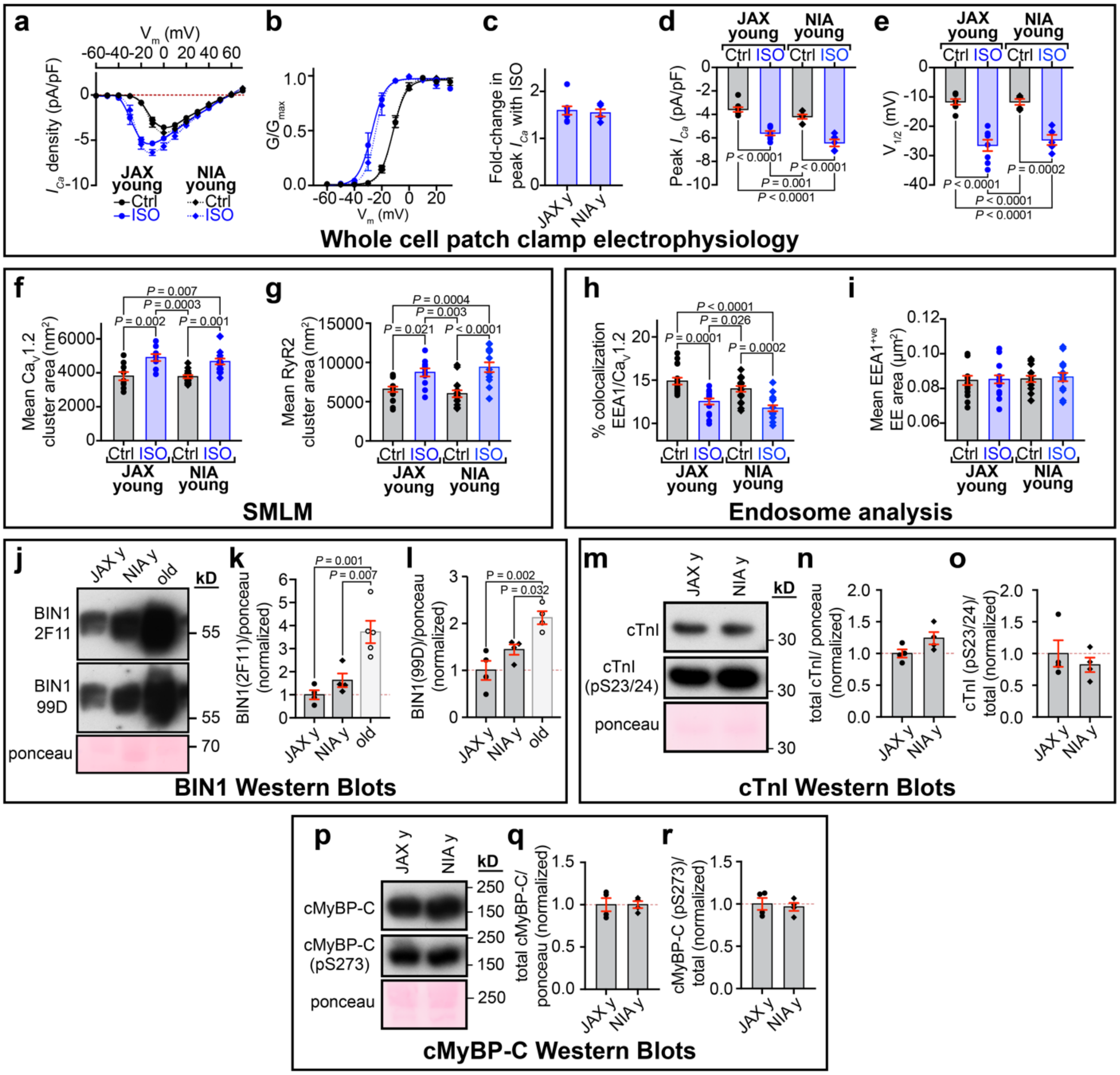


**Supplementary Figure 1. Comparison between young C57Bl/6 mice sourced from the Jackson Laboratory (JAX) and the National Institute on Aging (NIA)**. **a-e**, whole cell patch clamp electrophysiology data. **a**, plots showing the voltage dependence of $I_{Ca}$ density for JAX young ($N$ = 6, $n$ = 8) and NIA young ($N$ = 3, $n$ = 5) male mice. **b**, voltage-dependence of the normalized conductance ($G/G_{max}$) fit with Boltzmann functions. **c-e**, dot-plots showing fold change in $I_{Ca}$ with ISO (**c**), peak $I_{Ca}$ density (**d**), and $V_{1/2}$ of activation (**e**) for all groups. **f** and **g,** SMLM data showing dot-plots for mean $Ca_V1.2$ (**f**) and RyR2 (**g**) cluster areas for JAX young ($Ca_V1.2$:

control: $N = 3$, $n = 9$; ISO: $N = 3$, $n = 9$; RyR2: control: $N = 3$, $n = 17$; ISO: $N = 3$, $n = 11$) and NIA young ($Ca_V1.2$: control: $N = 3$, $n = 16$; ISO: $N = 3$, $n = 16$ ; RyR2: control: $N = 3$, $n = 15$; ISO: $N = 3$, $n = 15$). **h** and **i**, endosome analysis with dot-plots summarizing % colocalization between EEA1 and $Ca_V1.2$ (**h**), and EEA1 positive endosome areas (**i**) in JAX young (control: $N = 3$, $n = 16$; ISO: $N = 3$, $n = 16$) and NIA young (control: $N = 3$, $n = 17$; ISO: $N = 3$, $n = 16$) myocytes. **j-l**, BIN1 western blot analysis. **j**, western blot of BIN1 expression in whole heart lysates from JAX young, NIA young, and old mice probed with 2F11 (*top*) and 99D (*bottom*). Total ponceau was used for normalization. Histograms showing normalized BIN1 levels relative to JAX young for western blots probed with 2F11 (**k**) ($N = 4$-5, average of 4-6 replicates) and 99D (**l**) ($N = 4$, average of 2-3 replicates). **m-o**, cTnI western blot analysis. **m**, western blot of whole heart lysates from JAX-sourced young and NIA-sourced young mice showing total (*top*) and pS23/24 (*bottom*) cTnI expression. Total ponceau was used for normalization. Histograms showing normalized total (**n**) and pS23/24/total (**o**) cTnI levels relative to JAX-sourced young ($N = 4$, average of 3-4 replicates). **p-r**, cMyBP-C western blot analysis. **p**, western blot of whole heart lysates from JAX-sourced young and NIA-sourced young mice showing total (*top*) and pS273 (*bottom*) cMyBP-C expression. Total ponceau was used for normalization. Histograms showing normalized total (**q**) and pS273/total (**r**) cMyBP-C levels relative to JAX-sourced young ($N = 4$, average of 4 replicates). Whole cell data from JAX-sourced and NIA-sourced young mice is merged in Figs. 1b-f. SMLM data from NIA-sourced young myocytes in panels f and g is reproduced from Figs. 2b and 2d respectively. Endosomal JAX-sourced young data in panels h and i is reproduced from Figs. 4b and 4c respectively. Statistical analyses were performed using unpaired two-tailed Student's t-tests in c, n, o, q and r, two-way ANOVAs with multiple comparison post-hoc tests on d-i, and one-way ANOVAs on k and l with multiple comparison post-hoc tests. Source data are provided in the Source Data file and Uncropped Western Blots are provided at the end of this Supplementary Information file.

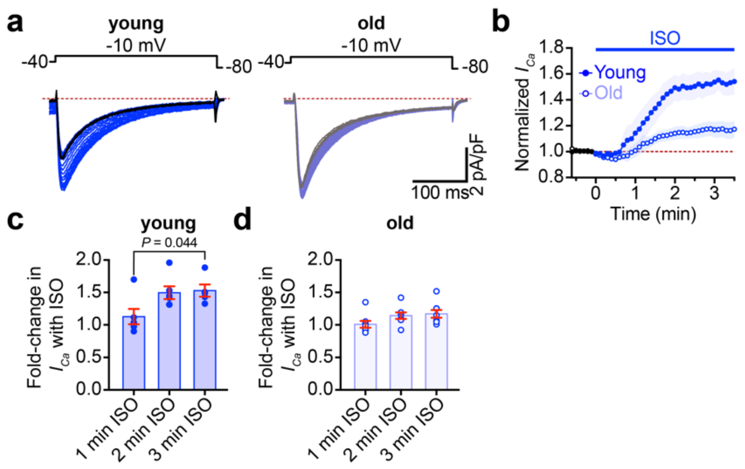


**Supplementary Figure 2. Time course of $I_{Ca}$ response to ISO in young and old myocytes. a**, representative perforated patch clamp currents elicited from young (*left*) and old (*right*) ventricular myocytes before (control; black traces) and during application of ISO (blue traces). **b**, diary plot of normalized $I_{Ca}$ density for young ($N$ = 3, $n$ = 6) and old ($N$ = 3, $n$ = 8) myocytes. **c** and **d**, dot-plots summarizing the time course of the fold-change in $I_{Ca}$ with ISO for young (**c**) and old (**d**) myocytes. Statistical analyses on data in c and d were performed using one-way ANOVAs with multiple comparison post-hoc tests. Young data in b and c is combination of data from NIA- and JAX-sourced young mouse myocytes. Note no significant differences were found in $I_{Ca}$ when NIA- and JAX-sourced young myocytes were compared (see Supplementary Fig. 1a-e). Data are presented as mean ± SEM. Source data are provided in the Source Data file.

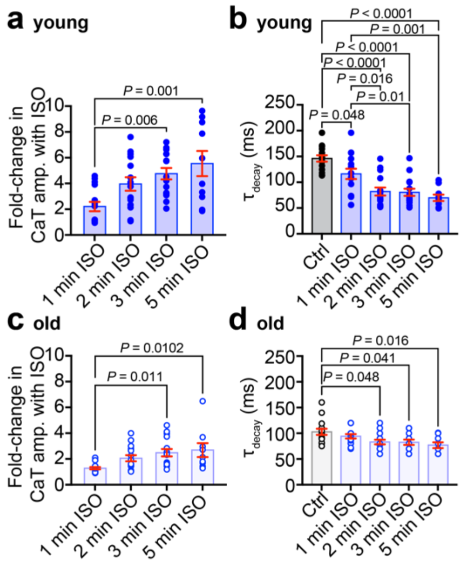


**Supplementary Figure 3. Time course of calcium transient response to ISO in young and old myocytes. a** and **c**, dot-plots showing the fold-increase in $Ca^{2+}$ transient amplitude after 1 min ($N$ = 5, $n$ = 15), 2 min ($N$ = 5, $n$ = 15), 3 min ($N$ = 5, $n$ = 15), and 5 min ($N$ = 4, $n$ = 10) of ISO in young myocytes (**a**) and 1 min ($N$ = 3, $n$ = 15), 2 min ($N$ = 3, $n$ = 15), 3 min ($N$ = 3, $n$ = 15), and 5 min ($N$ = 3, $n$ = 9) of ISO in old myocytes (**c**). **b** and **d**, dot-plots summarizing decay tau during the same time course in young (**b**) and old (**d**) myocytes. Statistical analyses in a-d were performed using one-way ANOVAs multiple comparison post-hoc tests. Young data in a and b is from NIA-sourced young mouse myocytes and the 3 min ISO data in a and c is reproduced from Fig. 1h. Data are presented as mean ± SEM. Source data are provided in the Source Data file.

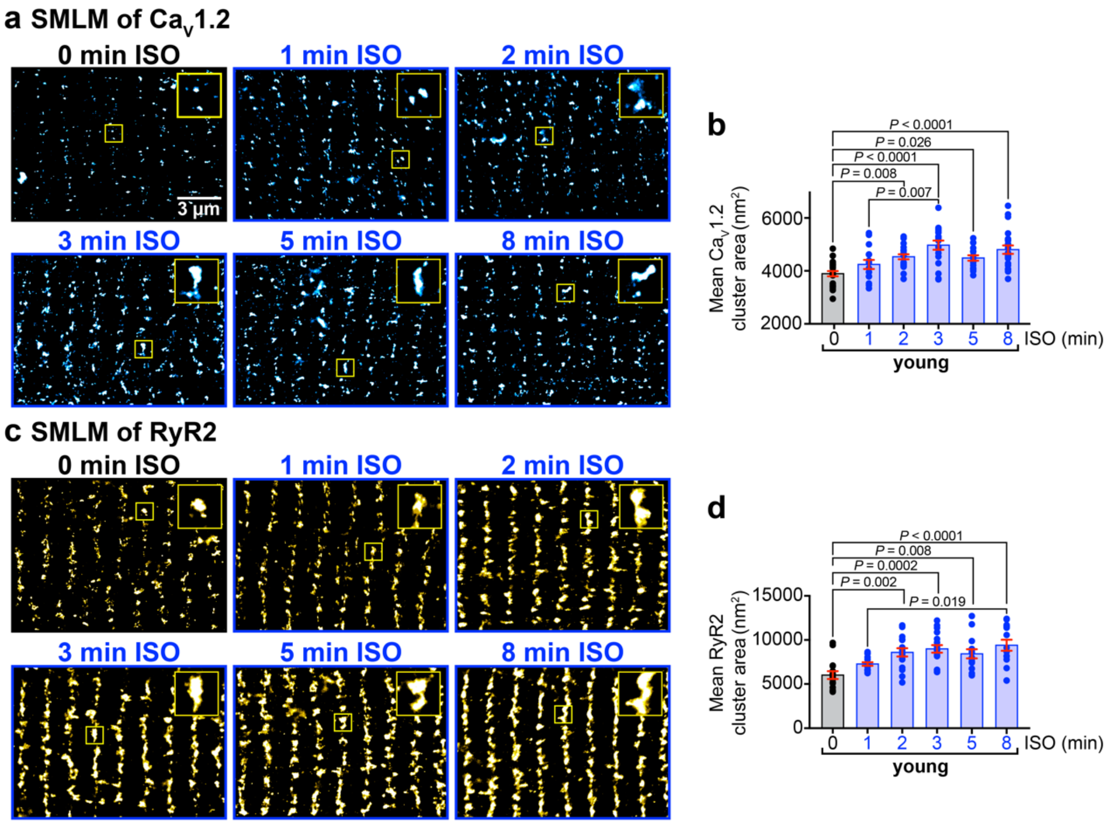


**Supplementary Figure 4**. **Time course of ISO-stimulated Ca$_V$1.2 and RyR2 clustering in young myocytes.** **a**, SMLM localization maps showing Ca$_V$1.2 channel localization and distribution in the t-tubules of young myocytes treated with 0, 1, 2, 3, 5 and 8 minutes of ISO. **b**, dot-plots summarizing the mean Ca$_V$1.2 cluster areas in young myocytes for the various time points (0 min ISO: $N$ = 3, $n$ = 22; 1 min ISO: $N$ = 3, $n$ = 15; 2 min ISO: $N$ = 3, $n$ = 20; 3 min ISO: $N$ = 3, $n$ = 18; 5 min ISO: $N$ = 3, $n$ = 17; 8 min ISO: $N$ = 3, $n$ = 22). **c** and **d**, show the same layout for RyR2 cluster areas in young myocytes (0 min ISO: $N$ = 3, $n$ = 15; 1 min ISO: $N$ = 3, $n$ = 16; 2 min ISO: $N$ = 3, $n$ = 16; 3 min ISO: $N$ = 3, $n$ = 18; 5 min ISO: $N$ = 3, $n$ = 14; 8 min ISO: $N$ = 3, $n$ = 15). Statistical analyses in b and d were performed using one-way ANOVAs with multiple comparisons post-hoc tests. Young data presented in b is pooled from NIA- and JAX-sourced young mouse myocytes and in d is exclusively from NIA-sourced young. Note there was no significant difference in Ca$_V$1.2 and RyR2 cluster areas when JAX- and NIA-sourced young mouse myocytes were compared (see Supplementary Fig. 1f-g). The NIA-sourced young data points in

panel b are reproduced from Fig. 2b, and the data in d is reproduced from Fig. 2d. Data are presented as mean ± SEM. Source data are provided in the Source Data file.

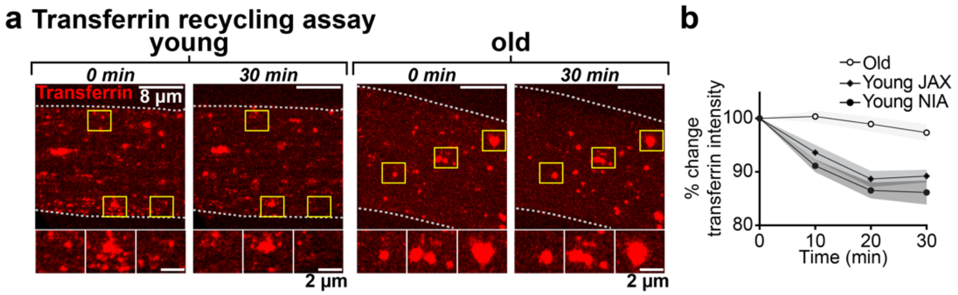


**Supplementary Figure 5. Transferrin receptor recycling is slowed in old myocytes. a**, representative images of young (*left*) and old (*right*) ventricular myocytes incubated with transferrin-Alexa 568 nm for 1 hr (time 0) and after washout (time 30 min). Yellow boxes show regions of interest zoomed-in below the images. **b**, quantification of transferrin-Alexa 568 nm % fluorescence change over a time course in young JAX-sourced ($N$ = 3, $n$ = 9), young NIA-sourced ($N$ = 3, $n$ = 9), and old ($N$ = 4, $n$ = 13) ventricular myocytes. Data are presented as mean ± SEM. Source data are provided in the Source Data file.

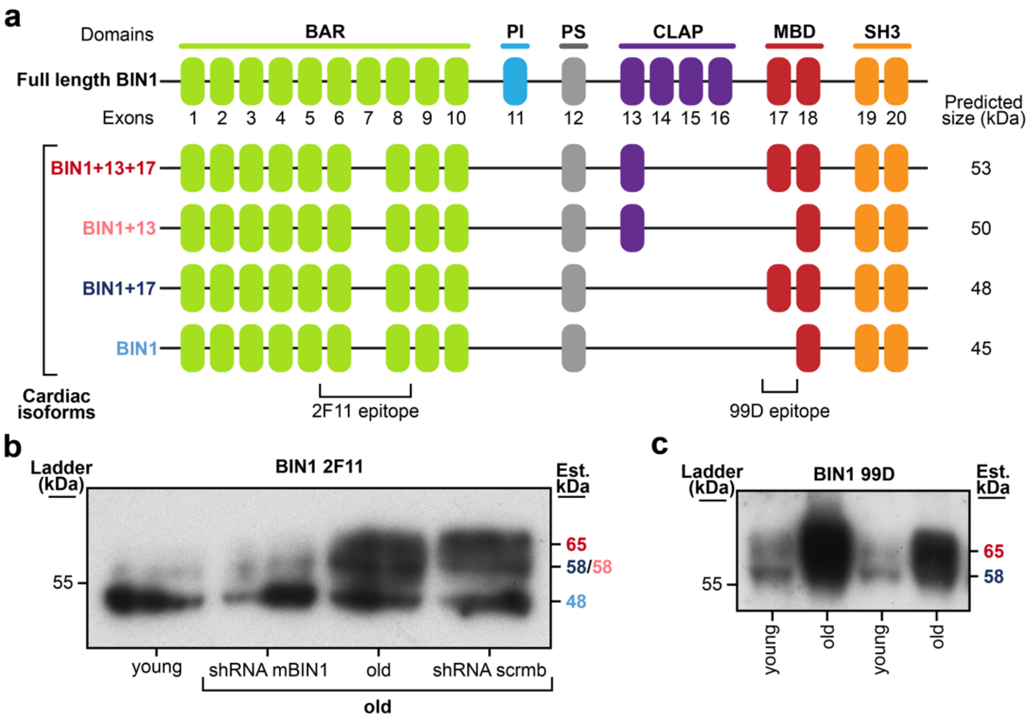


**Supplementary Figure 6. BIN1 isoforms present in the heart.** **a**, diagram illustrating the domains and exons present in full length BIN1 as well as the four isoforms present in the heart (BIN1, BIN1+13, BIN1+17, BIN1+13+17) with their respective predicted molecular weights. Antibody epitopes for 2F11 and 99D antibodies are shown on the bottom. **b,** example western blot showing the presence of three bands with 2F11, corresponding to BIN1, BIN1+13 and BIN1+17 combined, and BIN1+13+17. **c,** example western blot showing the presence of two bands with 99D, corresponding to BIN1+17 and BIN1+13+17. Source data are provided in the Source Data file and Uncropped Western Blots are provided at the end of this Supplementary Information file.

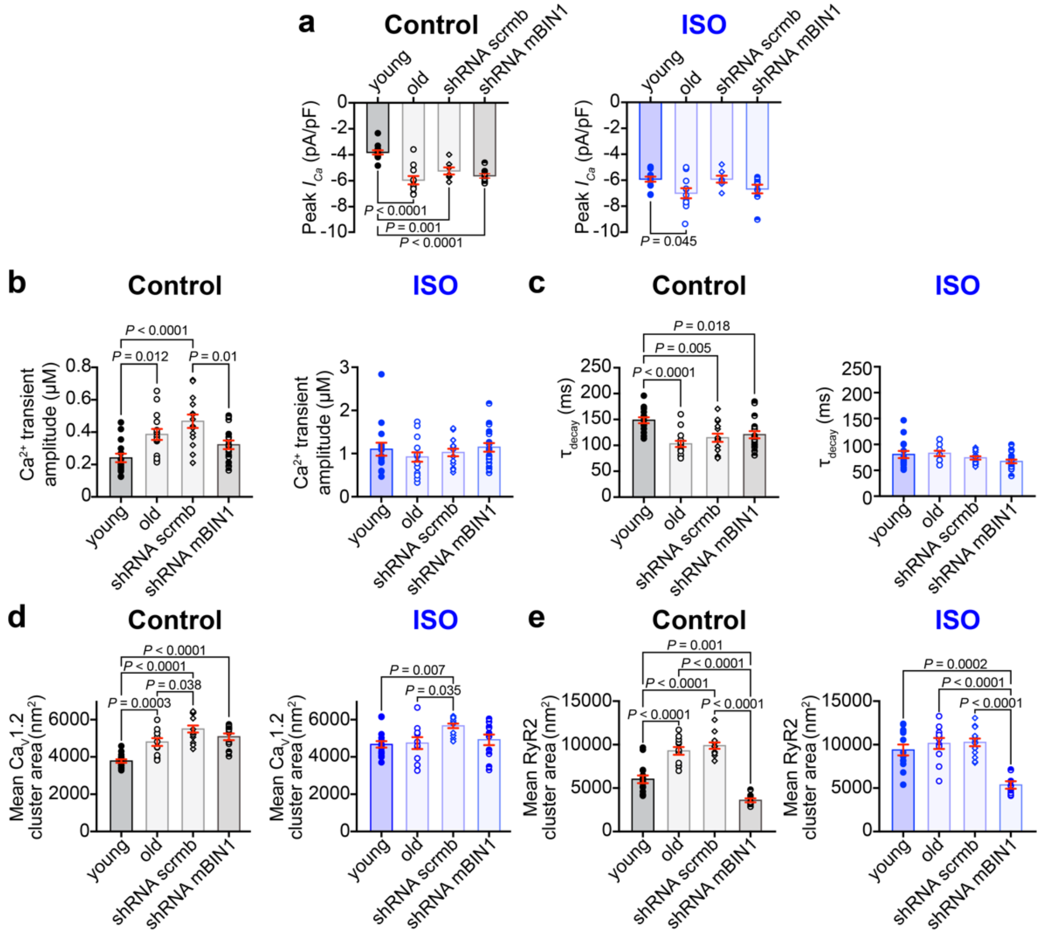


**Supplementary Figure 7. Further characterization of BIN1 knockdown in old mice. a**, dot-plots showing peak $I_{Ca}$ in control (left) and ISO (right) conditions for young, old, shRNA-scrmb (*N* = 3, *n* = 7) and shRNA-mBIN1 (*N* = 3, *n* = 9) transduced old myocytes. Old and young data points are reproduced from Fig. 1c. **b**, dot-plots summarizing $Ca^{2+}$ transient amplitude in control (left) and ISO (right) conditions from young, old, shRNA-scrmb (*N* = 3, *n* = 14) and shRNA-mBIN1 (*N* = 5, *n* = 18) transduced old myocytes. Old and young data points are reproduced from Fig. 1i. **c**, dot-plots showing $Ca^{2+}$ transient decay (ms) in control (*left*) and ISO (*right*) from young, old, shRNA-scrmb (*N* = 3, *n* = 14) and shRNA-mBIN1 (*N* = 5, *n* = 18) transduced old myocytes. Young and old data points are reproduced from Supplementary Fig. 3b and d. **d**, dot-plots summarizing the mean $Ca_V1.2$ channel cluster areas in control (left) and ISO (right) conditions for young, old, shRNA-scrmb (control: *N* = 3, *n* = 14; ISO: *N* = 3, *n* = 13) and shRNA-mBIN1 (control: *N* = 3, *n* =

12; ISO: $N$ = 3, $n$ = 11) transduced old myocytes. Old and young data points are reproduced from Fig. 2b. **e**, dot-plots summarizing the mean RyR2 channel cluster areas in control (left) and ISO (right) conditions for young, old, shRNA-scrmb (control: $N$ = 3, $n$ = 12; ISO: $N$ = 3, $n$ = 13) and shRNA-mBIN1 (control: $N$ = 3, $n$ = 9; ISO: $N$ = 3, $n$ = 8) transduced old myocytes. Old and young data points are reproduced from Fig. 2d. Statistical analyses were performed using one-way ANOVAs with multiple comparison post-hoc tests. Young data presented in a is a combination of data from NIA-sourced young and JAX-sourced young mouse myocytes, and in b-e is from NIA-sourced young myocytes. Data are presented as mean ± SEM. Source data are provided in the Source Data file.

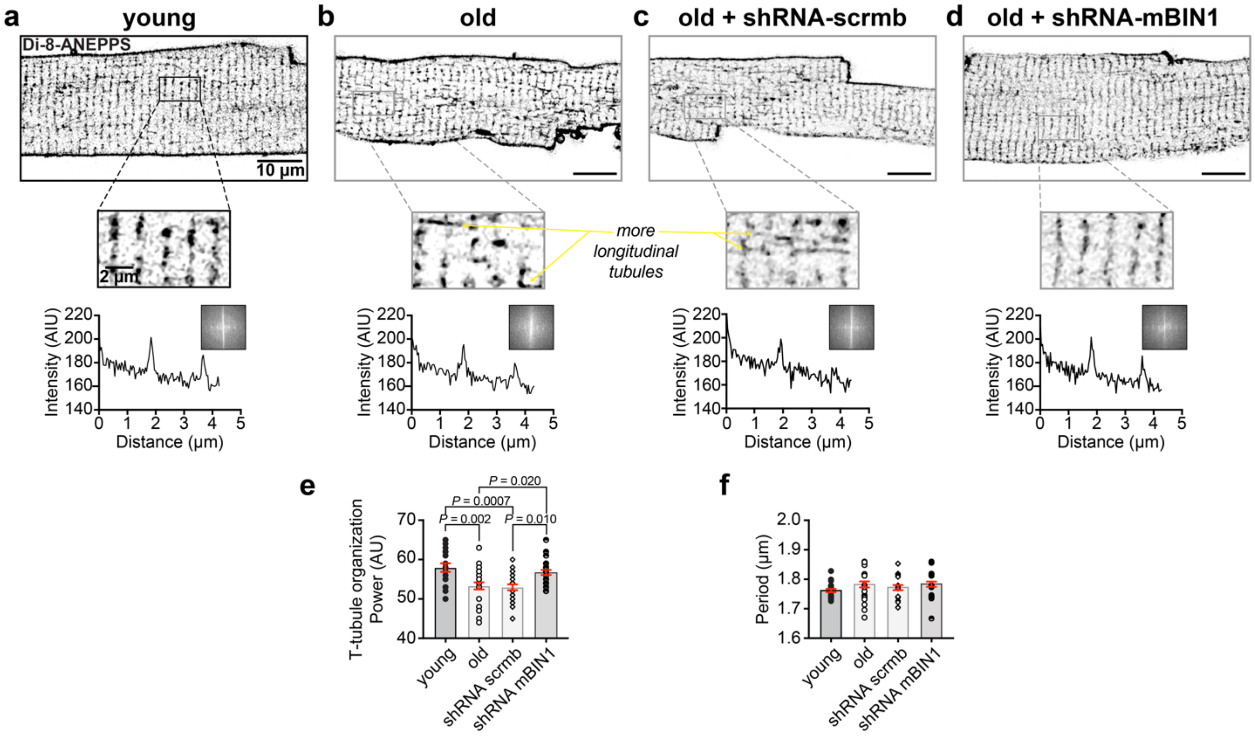


**Supplementary Figure 8. Youthful t-tubule network organization is recovered in old myocytes upon BIN1 knockdown. a-d**, representative images of t-tubules in cardiomyocytes labeled with di-8-ANEPPS isolated from young (**a**), old (**b**), old shRNA-scrmb transduced (**c**), and old shRNA-mBIN1 transduced (**d**) mice. Plotted below are graphs representing the power spectrum obtained from a fast fourier transform (FFT) analysis performed on the representative cells. **e-f:** Histograms representing **e**, t-tubule organization power (AIU) and **f,** periodicity (µm) for each group of young (*N* = 3, *n* = 20), old (*N* = 3, *n* = 26), old shRNA-scrmb (*N* = 3, *n* = 24) and old shRNA-mBIN1 (*N* = 3, *n* = 25). Statistical analyses on e and f were performed using one-way ANOVAs with multiple comparison post-hoc tests. Young data were obtained from NIA-sourced young mice. Data are presented as mean ± SEM. Source data are provided in the Source Data file.

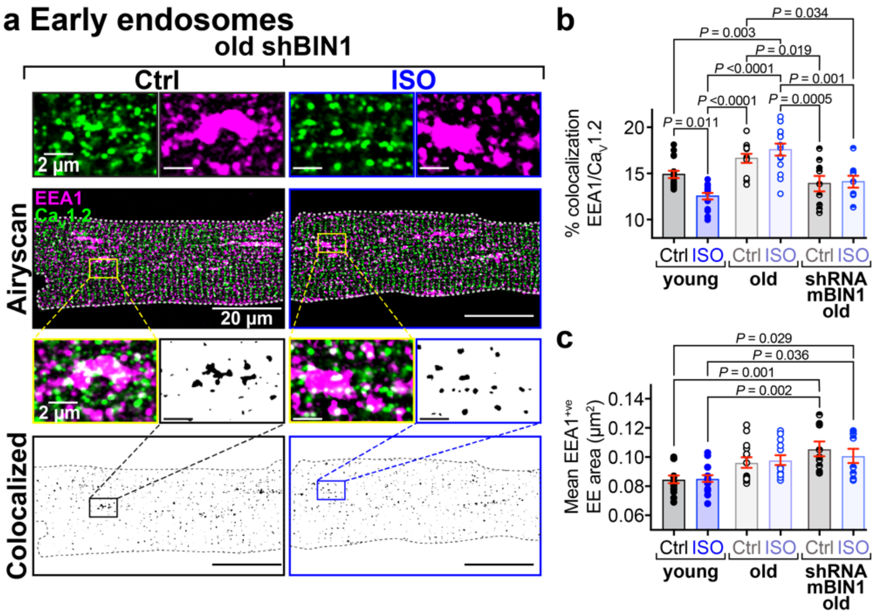


**Supplementary Figure 9. EEA1 positive early endosomes are not altered with BIN1 knockdown. a**, Airyscan super-resolution images of EEA1 (magenta) and $Ca_V1.2$ (green) immunostained shRNA-mBIN1 transduced old myocytes under control and ISO-stimulated conditions. *Bottom:* Binary colocalization map showing overlapped expression. **b**, dot-plots summarizing % colocalization between EEA1 and $Ca_V1.2$, and **c**, mean area of EEA1 positive endosomes in young, old and shRNA-mBIN1 (control: $N$ = 3, $n$ = 9; ISO: $N$ = 3, $n$ = 9) transduced old myocytes. Old and young data points are reproduced from Fig. 4b and c. Statistical analyses in b and c were performed using two-way ANOVAs with multiple comparison post-hoc tests. Young data presented is from JAX-sourced young mice. Note no significant differences in EEA1/$Ca_V1.2$ colocalization, responsivity to ISO, or endosome size was detected when young JAX-sourced and young NIA-sourced mice were compared (see Supplementary Fig. 1h-i). Data are presented as mean ± SEM. Source data are provided in the Source Data file.

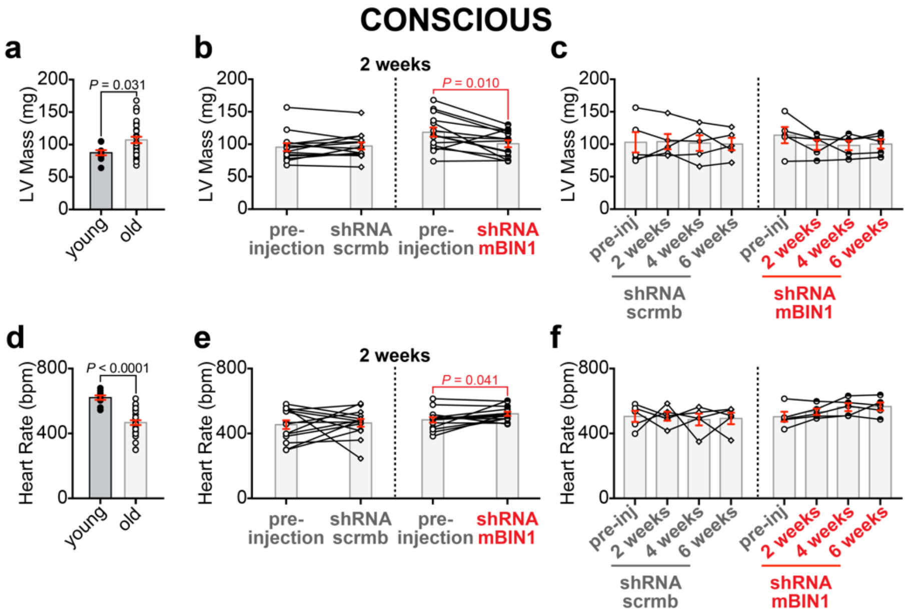


**Supplementary Figure 10. Additional data from conscious mice using M-mode echocardiography.** Summary dot-plots for conscious young (*N* = 10) and old (*N* = 30) mice, paired results before and two weeks after RO-injection of old mice with shRNA-scrmb (*N* = 14) and shRNA-mBIN1 (*N* = 14), and paired results before and after two, four and six weeks RO-injection of old mice with shRNA-scrmb (*N* = 5) and shRNA-mBIN1 (*N* = 5) for the following measurements are displayed: **a-c**, left ventricular (LV) mass and **d-f**, heart rate. Unpaired two-tailed Student's t-tests were performed on data displayed in a and d. Paired two-tailed Student's t-tests were performed on data displayed in b and e. One-way ANOVAs with multiple comparisons post-hoc tests were performed on data displayed in c and f. Young data presented is from NIA-sourced young mice. Data are presented as mean ± SEM. Source data are provided in the Source Data file.

# CONSCIOUS Dimensions

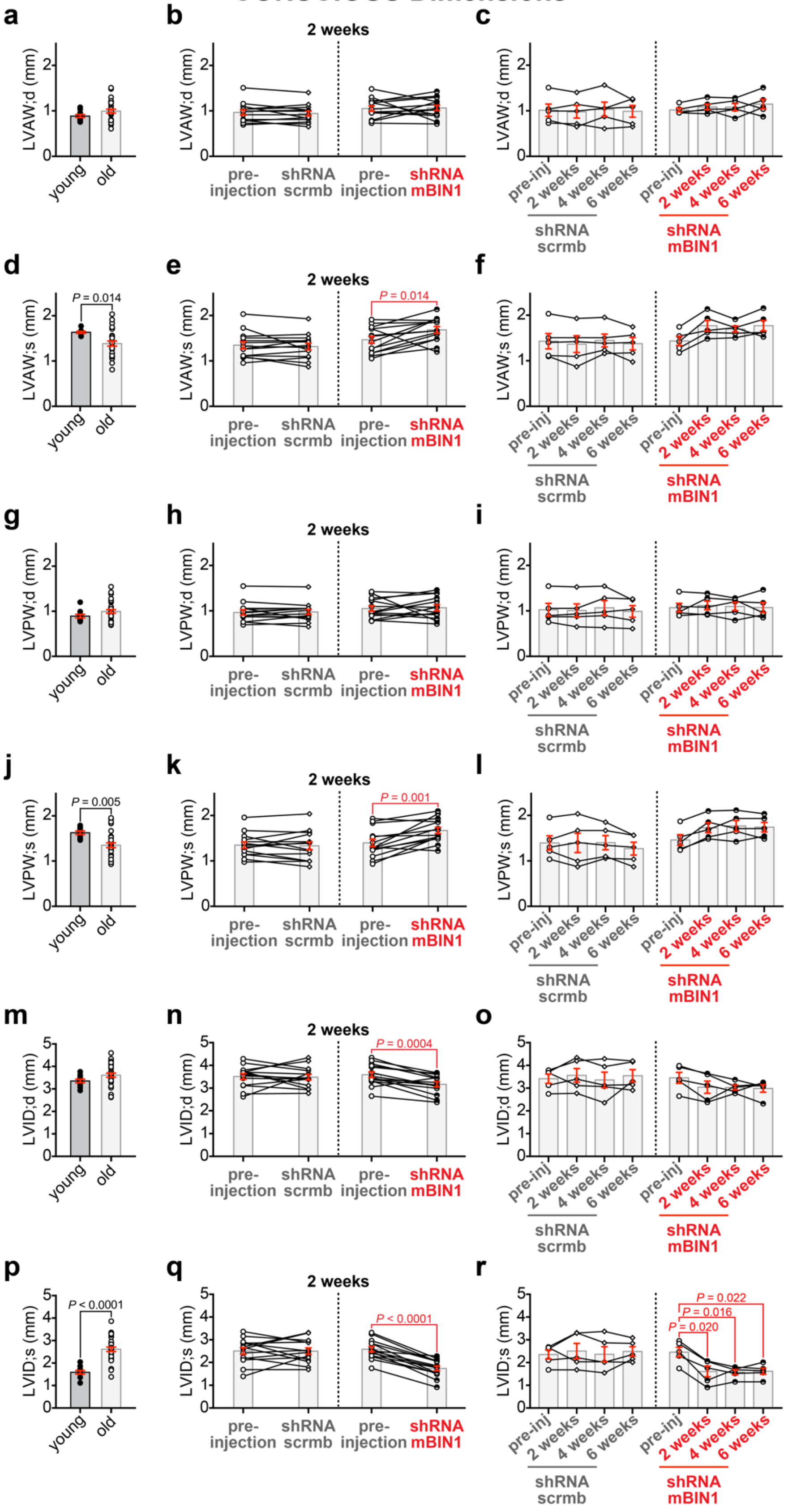

**Supplementary Figure 11. Left ventricular dimensions of conscious young and old mice from M-mode echocardiography.** Summary dot-plots for conscious young (*N* = 10) and old (*N* = 30) mice, paired results before and two weeks after RO-injection of old mice with shRNA-scrmb (*N* = 14) and shRNA-mBIN1 (*N* = 14), and paired results before and after two, four and six weeks RO-injection of old mice with shRNA-scrmb (*N* = 5) and shRNA-mBIN1 (*N* = 5) for the following measurements are displayed: **a**-**c**, left ventricular anterior wall in diastole (LVAW;d), **d**-**f**, left ventricular anterior wall in systole (LVAW;s), **g**-**i**, left ventricular posterior wall in diastole (LVPW;d), **j**-**l**, left ventricular posterior wall in systole (LVPW;s), **m**-**o**, left ventricular inner diameter in diastole (LVID;d), **p**-**r**, left ventricular inner diameter in systole (LVID;s). Unpaired two-tailed Student's t-tests were performed on data displayed in a, d, g, j, m and p. Paired two-tailed Student's t-tests were performed on data displayed in b, e, h, k, n and q. One-way ANOVAs with multiple comparisons post-hoc test were performed on data displayed in c, f, i, l, o and r. Young data presented is from NIA-sourced young mice. Data are presented as mean ± SEM. Source data are provided in the Source Data file.

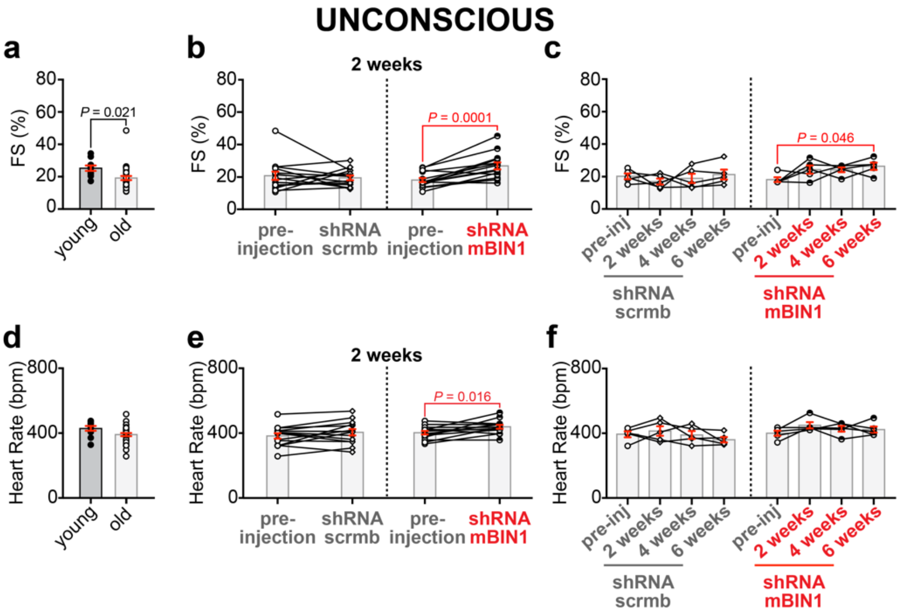


**Supplementary Figure 12. Additional data from unconscious mice using M-mode echocardiography.** Summary dot-plots for unconscious young (*N* = 10) and old (*N* = 30) mice, paired results before and two weeks after RO-injection of old mice with shRNA-scrmb (*N* = 14) and shRNA-mBIN1 (*N* = 14), and paired results before and after two, four and six weeks RO-injection of old mice with shRNA-scrmb (*N* = 5) and shRNA-mBIN1 (*N* = 5) for the following measurements are displayed: **a-c**, fractional shortening (FS) and **d-f**, heart rate. Unpaired two-tailed Student's t-tests were performed on data displayed in a and d. Paired two-tailed Student's t-tests were performed on data displayed in b and e. One-way ANOVAs with multiple comparisons post-hoc test were performed on data displayed in c and f. Young data presented is from NIA-sourced young mice. Data are presented as mean ± SEM. Source data are provided in the Source Data file.

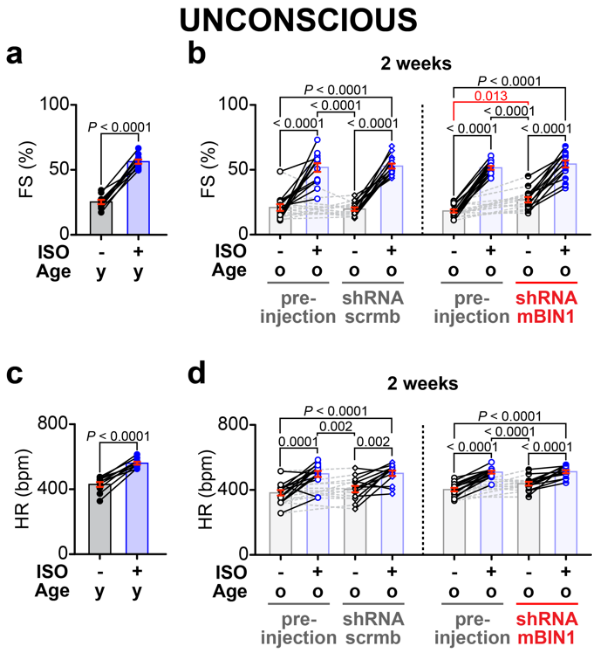


**Supplementary Figure 13. Analysis of systolic function in unconscious mice with and without ISO.** Summary dot-plots for unconscious young mice (*N* = 10), and paired results before and two weeks after RO-injection of old mice with shRNA-scrmb (*N* = 14) and shRNA-mBIN1 (*N* = 14), before and after intraperitoneal injection of 0.1 mg/kg ISO for the following measurements are displayed: **a** and **b**, fractional shortening (FS) and **c** and **d**, heart rate (HR). Paired two-tailed Student's t-tests were performed on data displayed in a and c. Two-way ANOVAs with multiple comparison post-hoc tests were performed on data displayed in b and d. Young data presented is from NIA-sourced young mice. Data are presented as mean ± SEM. Source data are provided in the Source Data file.

| Group | (*N*, *n*) | Fold-change in peak $I_{Ca}$ with ISO | Capacitance (pF) | $V_{1/2}$ (mV) | | Slope factor | |
|---|---|---|---|---|---|---|---|
| | | | | Control | ISO | Control | ISO |
| **young** | $N = 9$, $n = 13$ | 1.57 ± 0.06 | 157.8 ± 9.5 | -11.7 ± 0.7 | -25.8 ± 1.3 $^{P < 0.0001}$ | 4.6 ± 0.3 | 3.3 ± 0.3 $^{P = 0.0008}$ |
| **old** | $N = 5$, $n = 11$ | 1.19 ± 0.06 $^{P = 0.0001}$ | 153.3 ± 19.9 | -21.1 ± 1.0 | -27.9 ± 1.1 $^{P < 0.0001}$ | 3.9 ± 0.2 | 3.3 ± 0.2 $^{P = 0.0002}$ |
| **shRNA-scrmb old** | $N = 3$, $n = 7$ | 1.14 ± 0.06 $^{P = 0.0002}$ | 162.1 ± 20.4 | -20.8 ± 2.6 | -27.9 ± 2.0 $^{P = 0.0007}$ | 4.9 ± 0.5 | 4.1 ± 0.5 $^{P = 0.0318}$ |
| **shRNA-mBIN1 old** | $N = 3$, $n = 9$ | 1.19 ± 0.06 $^{P = 0.0003}$ | 168.7 ± 12.9 | -21.2 ± 1.0 | -30.4 ± 1.0 $^{P < 0.0001}$ | 4.0 ± 0.3 | 3.0 ± 0.3 $^{P = 0.0005}$ |

**Supplementary Table 1. ISO-stimulated changes in peak $I_{Ca}$ and voltage dependence of $G/G_{max}$ of young and old ventricular cardiomyocytes.** Mean ± SEM for the fold-change in peak $I_{Ca}$ (a one-way ANOVA with a multiple comparison post-hoc test was used to compare the four groups; p-values indicates significant difference from young mice), the whole cell capacitance, and for the $V_{1/2}$ and slope factor of $G/G_{max}$ fits (paired two-tailed Student's t-test; p-values indicates significant difference between ISO values and their respective controls). This data is also highlighted in Figs. 1 and 6, and Supplementary Fig. 10. Young data in this table is pooled from NIA-sourced and JAX-sourced young myocytes. Note there was no significant difference in $I_{Ca}$ when data obtained from NIA- and JAX-sourced young mice were compared (see Supplementary Fig. 1a-e). Data are presented as mean ± SEM. Source data are provided in the Source Data file.

## Uncropped Western Blots

### Figure 5a

Green boxes represent approximate areas used for analysis.
Blue boxes represent areas used for representative images in the figures.
Some membranes were cut in order to blot different proteins simultaneously.

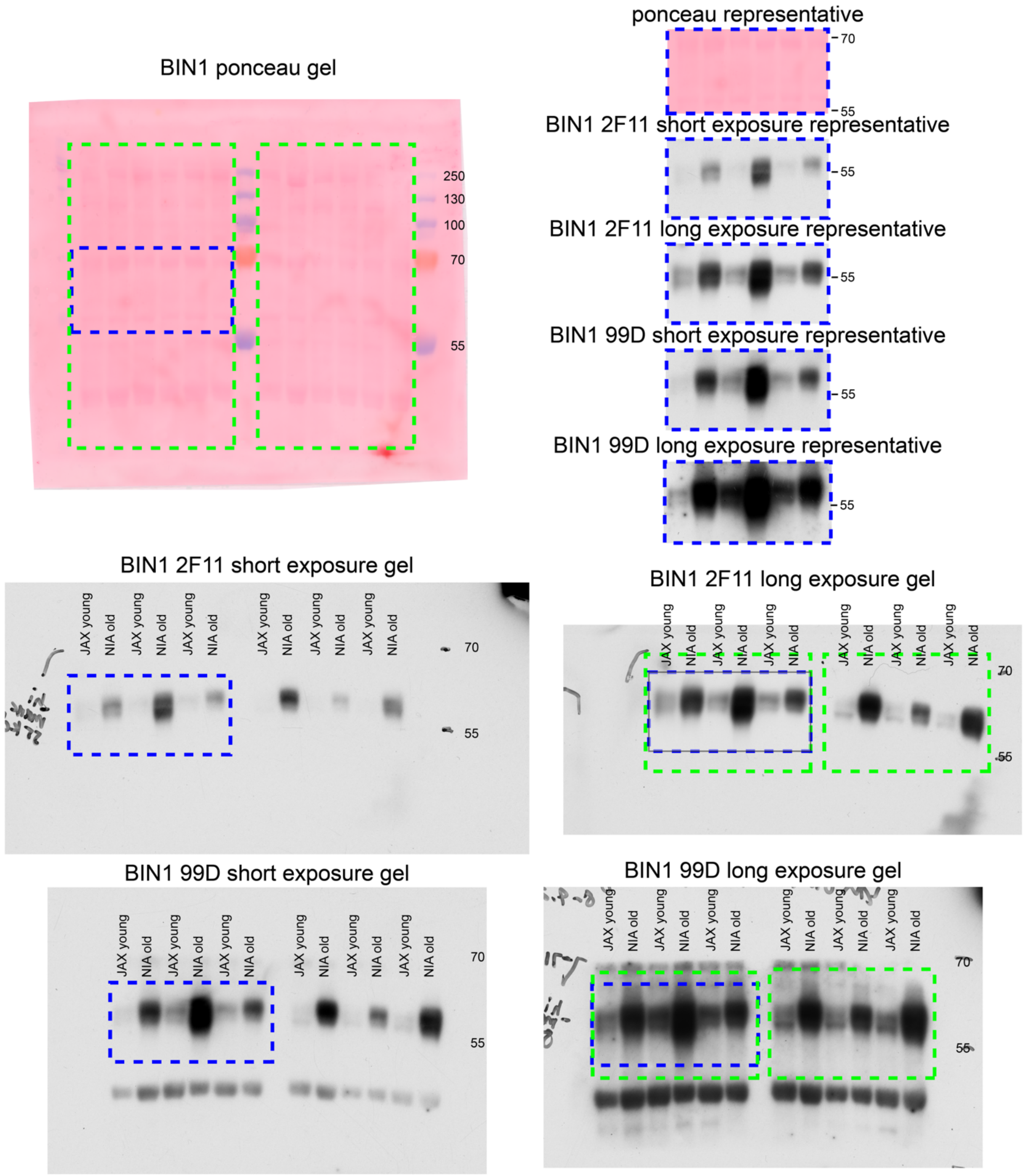

**Figure 5e**

Green boxes represent approximate areas used for analysis.
Blue boxes represent areas used for representative images in the figures.
Some membranes were cut in order to blot different proteins simultaneously.

BIN1 ponceau gel

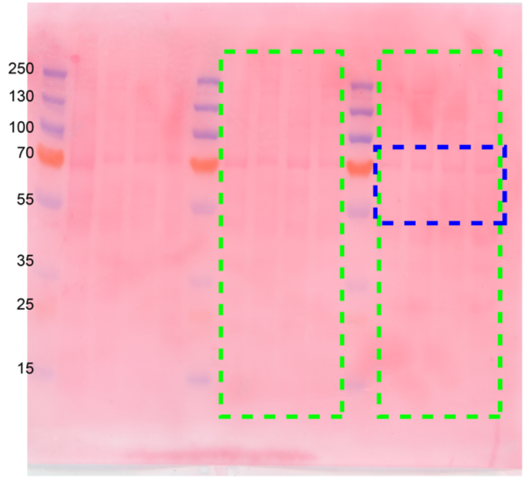


BIN1 2F11 short exposure representative

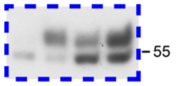


BIN1 2F11 long exposure representative

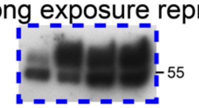


ponceau representative

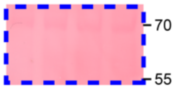


BIN1 2F11 short exposure gel

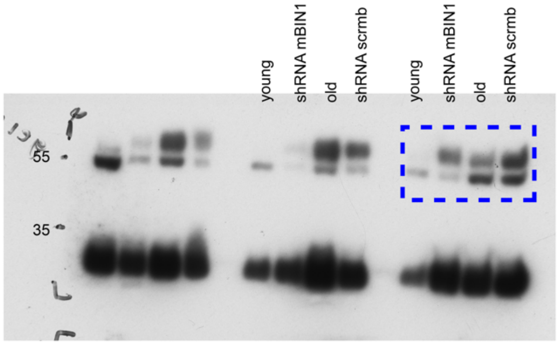


BIN1 2F11 long exposure gel

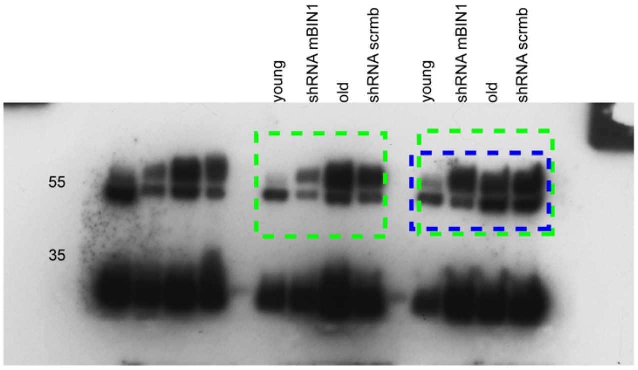

**Figure 9a**

Green boxes represent approximate areas used for analysis.
Blue boxes represent areas used for representative images in the figures.
Some membranes were cut in order to blot different proteins simultaneously.

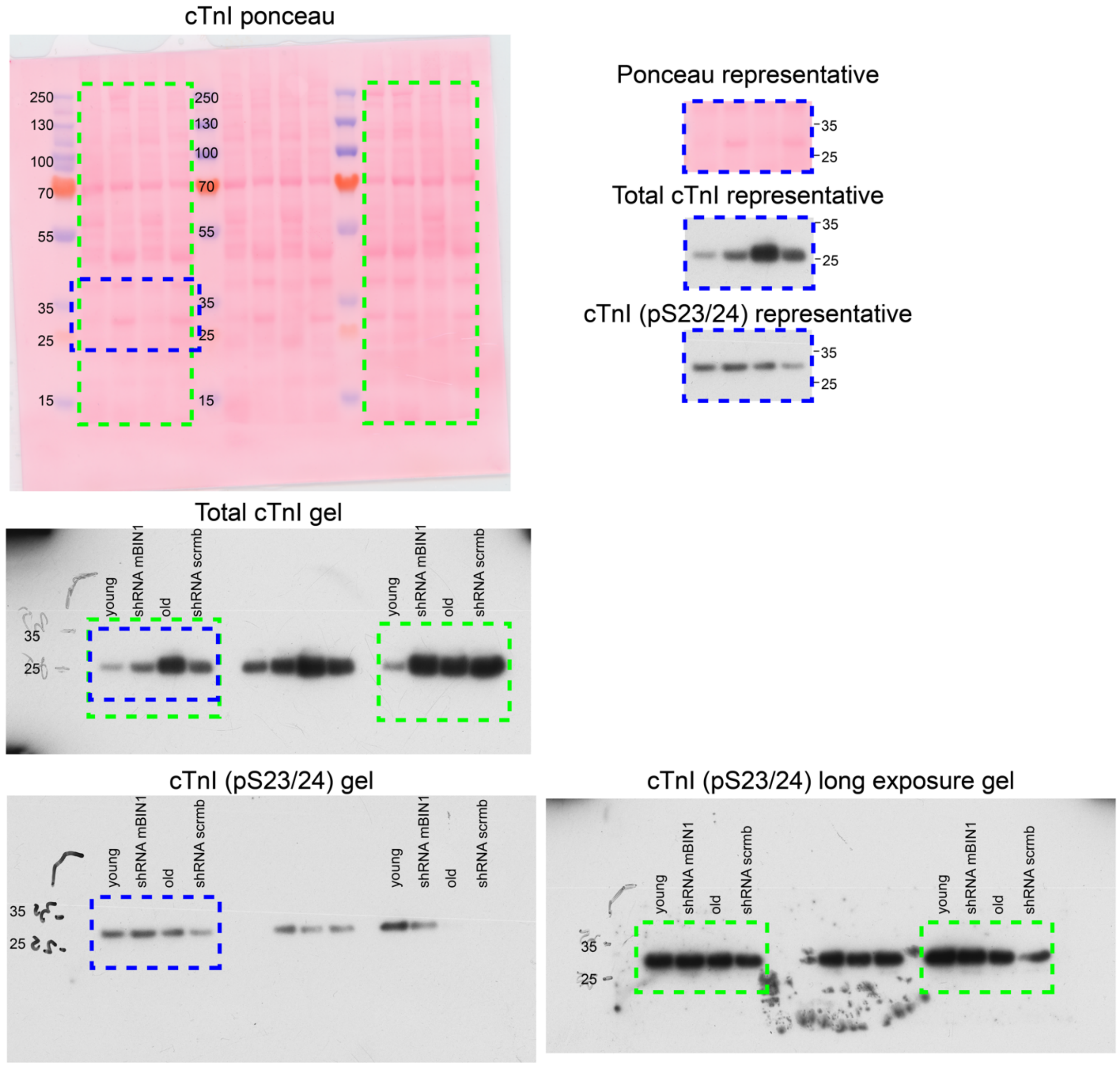

**Figure 9d**

Green boxes represent approximate areas used for analysis.
Blue boxes represent areas used for representative images in the figures.
Some membranes were cut in order to blot different proteins simultaneously.

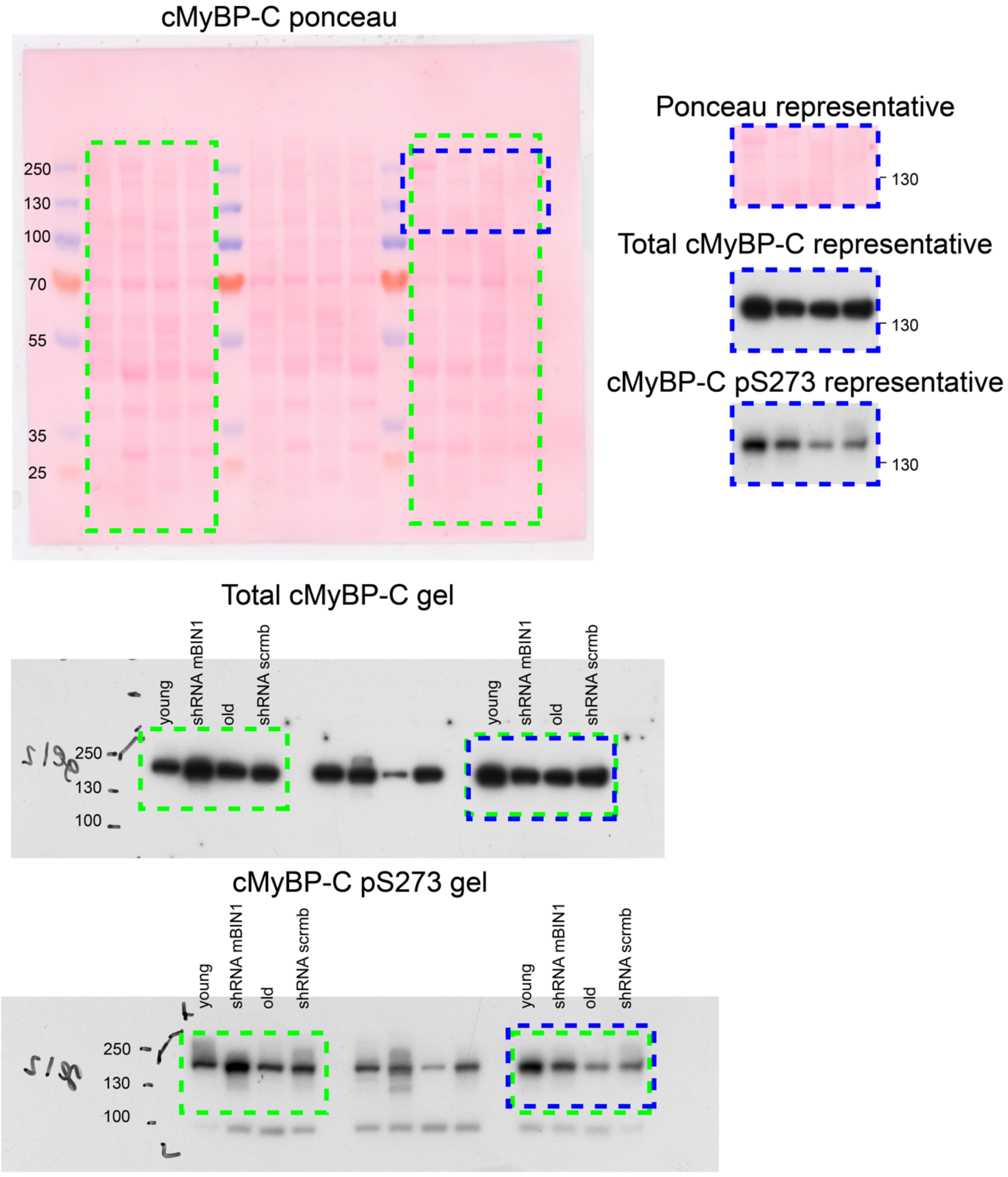

**Figure S1j**

Green boxes represent approximate areas used for analysis.
Blue boxes represent areas used for representative images in the figures.
Some membranes were cut in order to blot different proteins simultaneously.

BIN1 ponceau gel

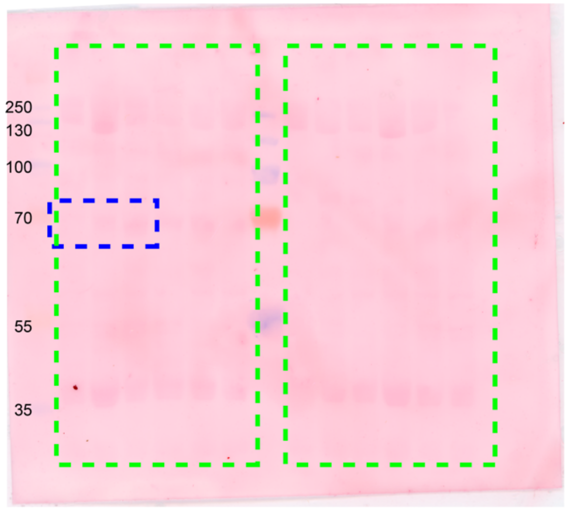


NIA vs JAX BIN1 ponceau representative

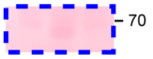


NIA vs JAX BIN1 2F11 representative

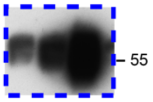


NIA vs JAX BIN1 99D representative

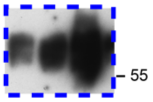


BIN1 2F11 gel

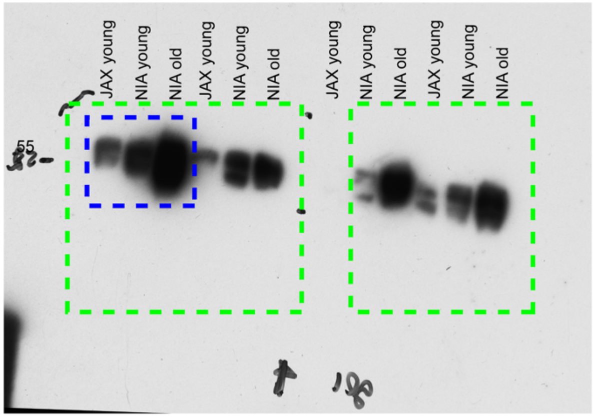


BIN1 99D gel

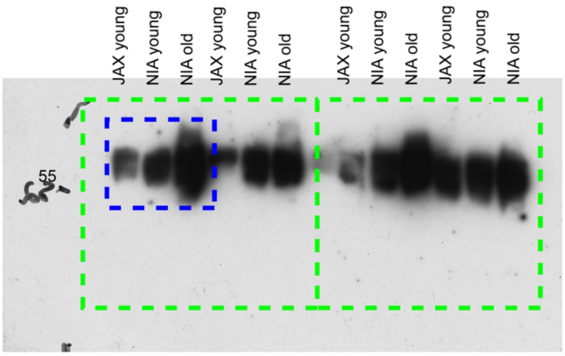

**Figure S1m**

Green boxes represent approximate areas used for analysis.
Blue boxes represent areas used for representative images in the figures.
Some membranes were cut in order to blot different proteins simultaneously.

cTnI ponceau gel

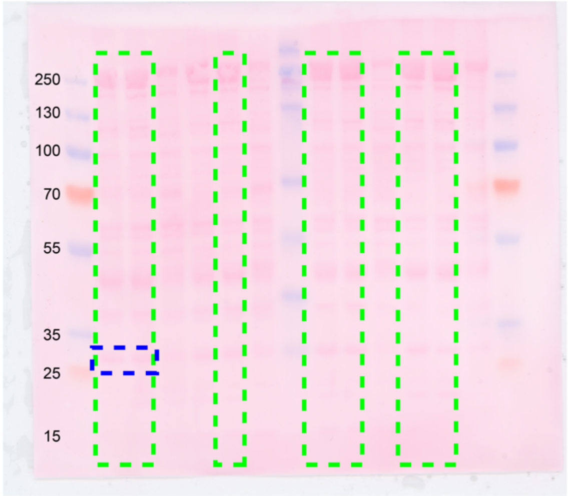


NIA vs JAX ponceau representative

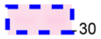


NIA vs JAX cTnI (pS23/24) representative

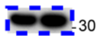


NIA vs JAX total cTnI representative

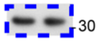


cTnI (pS23/24) gel

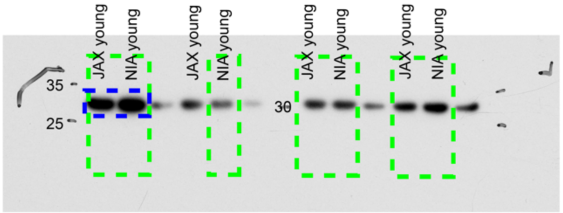


Total cTnI Gel

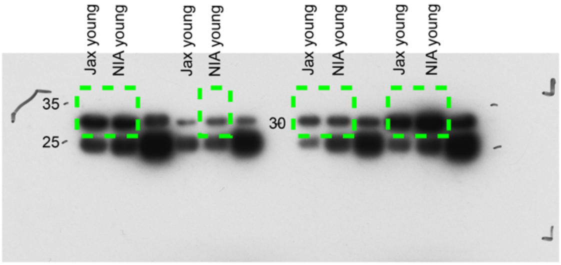


Total cTnI Gel - Lighter Exposure

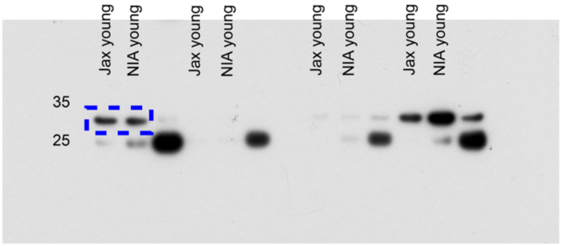

**Figure S1p**

Green boxes represent approximate areas used for analysis.
Blue boxes represent areas used for representative images in the figures.
Some membranes were cut in order to blot different proteins simultaneously.

cMyBP-C ponceau gel

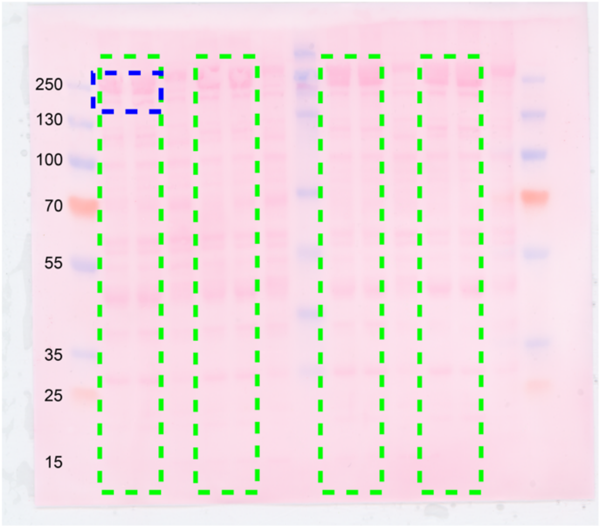


NIA vs JAX ponceau representative

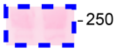


NIA vs JAX cMyBP-C pS273 representative

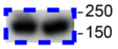


NIA vs JAX total cMyBP-C representative

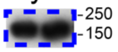


cMyBP-C pS273 gel

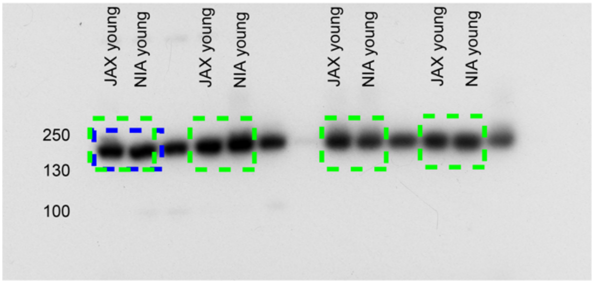


Total cMyBP-C gel

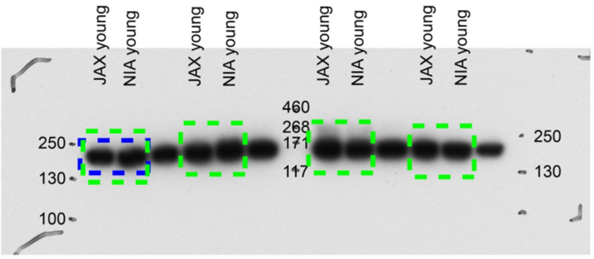

**Figure S6b**

Blue boxes represent areas used for representative images in the figures.
Some membranes were cut in order to blot different proteins simultaneously.

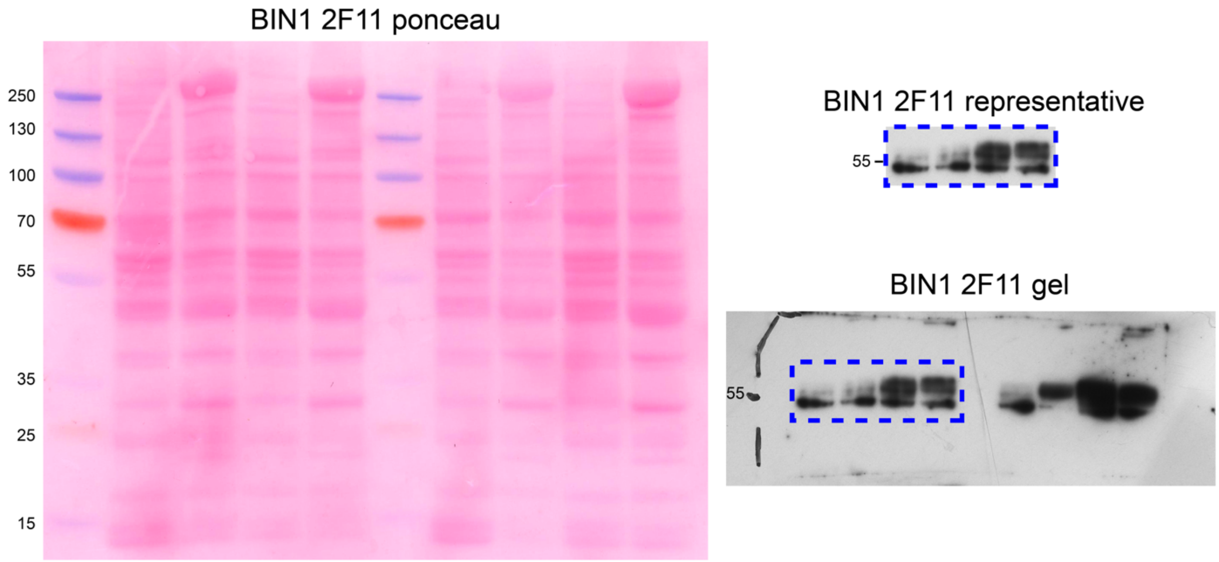


**Figure S6c**

Blue boxes represent areas used for representative images in the figures.
Some membranes were cut in order to blot different proteins simultaneously.

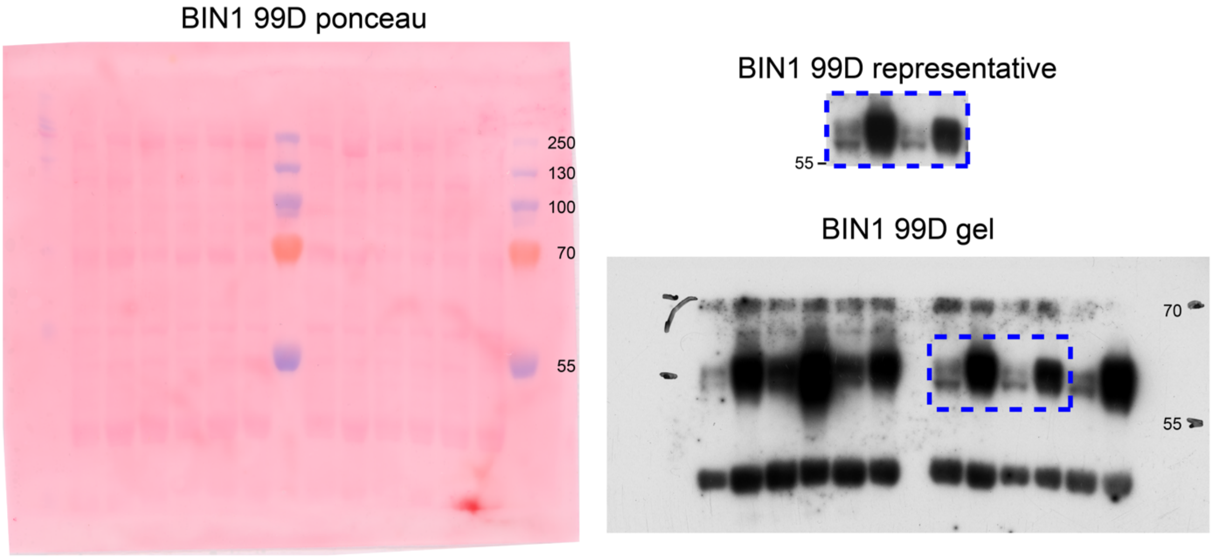

Supplement: Supplementary file 1 — Supplementary Information [file 41467_2024_47847_MOESM1_ESM.pdf]
