# Supplementary material for: BIN1 knockdown rescues systolic dysfunction in aging male mouse hearts
Source: Nat Commun. 2024 Apr 25;15:3528. doi: 10.1038/s41467-024-47847-8 (PMC11045846; doi:10.1038/s41467-024-47847-8)
Supplement: Supplementary file 3 — Description of additional supplementary files [file 41467_2024_47847_MOESM3_ESM.pdf]

Description of Additional Supplementary Files

Supplementary Movie 1: ISO-stimulated Ca_V1.2 dynamics are reduced in old myocytes compared to young. Videos showing TIRF time series recorded from representative young (JAX; left) and old (right) AAV9-Ca_Vβ_{2a}-paGFP transduced ventricular myocytes before and during β-AR activation with 100 nM ISO. Videos were sped up to ~12x (from the recorded 10.34 fps to 124 fps) for display purposes.
