# Supplementary material for: BIN1 knockdown rescues systolic dysfunction in aging male mouse hearts
Source: Nat Commun. 2024 Apr 25;15:3528. doi: 10.1038/s41467-024-47847-8 (PMC11045846; doi:10.1038/s41467-024-47847-8)
Supplement: Supplementary file 5 — Reporting Summary [file 41467_2024_47847_MOESM5_ESM.pdf]

nature portfolio

Corresponding author(s): Rose E Dixon

Last updated by author(s): 04/12/2024

Reporting Summary

Nature Portfolio wishes to improve the reproducibility of the work that we publish. This form provides structure for consistency and transparency in reporting. For further information on Nature Portfolio policies, see our Editorial Policies and the Editorial Policy Checklist.

Statistics

For all statistical analyses, confirm that the following items are present in the figure legend, table legend, main text, or Methods section.

n/a	Confirmed	
☐	☒	The exact sample size (*n*) for each experimental group/condition, given as a discrete number and unit of measurement
☐	☒	A statement on whether measurements were taken from distinct samples or whether the same sample was measured repeatedly
☐	☒	The statistical test(s) used AND whether they are one- or two-sided<br>*Only common tests should be described solely by name; describe more complex techniques in the Methods section.*
☐	☒	A description of all covariates tested
☐	☒	A description of any assumptions or corrections, such as tests of normality and adjustment for multiple comparisons
☐	☒	A full description of the statistical parameters including central tendency (e.g. means) or other basic estimates (e.g. regression coefficient) AND variation (e.g. standard deviation) or associated estimates of uncertainty (e.g. confidence intervals)
☐	☒	For null hypothesis testing, the test statistic (e.g. *F*, *t*, *r*) with confidence intervals, effect sizes, degrees of freedom and *P* value noted<br>*Give P values as exact values whenever suitable.*
☒	☐	For Bayesian analysis, information on the choice of priors and Markov chain Monte Carlo settings
☒	☐	For hierarchical and complex designs, identification of the appropriate level for tests and full reporting of outcomes
☒	☐	Estimates of effect sizes (e.g. Cohen's *d*, Pearson's *r*), indicating how they were calculated

Our web collection on statistics for biologists contains articles on many of the points above.

Software and code

Policy information about availability of computer code

Data collection	All software utilized for data collection in this study is commercially available. The specific software used is listed below:<br>Electrophysiology - Clampex (pClamp version: 10.6.2.2; Molecular Devices, San Jose, CA, USA)<br>TIRF imaging - Metamorph (version: 7.10.1.161; Molecular Devices)<br>Airyscan and confocal line scan imaging- Zen Microscopy Software (version: 14.0.27.201; Zeiss Group, Oberkochen, Germany)<br>Single molecule localization microscopy - LAS X (version: 3.7.5.24914; Leica Microsystems, Deerfield, IL, USA)<br>Imaging of western blot membranes: Medical Film Processor (SRX-lOlA, Konica Minolta, Tokyo, Japan); Sapphire Biomolecular lmager, (Azure Biosystems, Inc, Dublin California)<br>Echocardiography - Vevo 2100 Imaging system (VisualSonics, Fujifilm, Toronto, ON, Canada)<br>Quantitative RT-PCR - Applied Biosystems ViiA Real-Time PCR system (Applied Biosystems, Waltham, MA)
Data analysis	TIRF, confocal and SMLM data was analyzed with lmageJ/FIJI (NIH); Electrophysiology data was analyzed with Clampfit (pClamp version 10.6.2.2; Molecular Devices).<br>Quantification of protein expression on western blots was performed using Adobe Photoshop (Creative Cloud version 5.9.0.373, Photoshop Version 23.4.1) or lmageJ/FIJI (version 2.3.0/l.53q)<br>Echocardiography was analyzed using Vevo 2100 Imaging system (VisualSonics, Fujifilm, Toronto, ON, Canada).<br>Quantitative RT-PCR was analyzed using Applied Biosystems ViiA Real-Time PCR system (Applied Biosystems, Waltham, MA)<br>All graphing and statistics were performed in Graph pad Prism (version 9 for Mac; Graph Pad Software, San Diego, California USA).

For manuscripts utilizing custom algorithms or software that are central to the research but not yet described in published literature, software must be made available to editors and reviewers. We strongly encourage code deposition in a community repository (e.g. GitHub). See the Nature Portfolio guidelines for submitting code & software for further information.

Data

Policy information about availability of data

All manuscripts must include a data availability statement. This statement should provide the following information, where applicable:

- Accession codes, unique identifiers, or web links for publicly available datasets
- A description of any restrictions on data availability
- For clinical datasets or third party data, please ensure that the statement adheres to our policy

Data generated in this study are provided in the Source Data file and Supplementary Information.

Research involving human participants, their data, or biological material

Policy information about studies with human participants or human data. See also policy information about sex, gender (identity/presentation), and sexual orientation and race, ethnicity and racism.

Reporting on sex and gender	N/A
Reporting on race, ethnicity, or other socially relevant groupings	N/A
Population characteristics	N/A
Recruitment	N/A
Ethics oversight	N/A

Note that full information on the approval of the study protocol must also be provided in the manuscript.

Field-specific reporting

Please select the one below that is the best fit for your research. If you are not sure, read the appropriate sections before making your selection.

☒ Life sciences ☐ Behavioural & social sciences ☐ Ecological, evolutionary & environmental sciences

For a reference copy of the document with all sections, see nature.com/documents/nr-reporting-summary-flat.pdf

Life sciences study design

All studies must disclose on these points even when the disclosure is negative.

Sample size	We did not perform an a priori sample size calculation. However, all experimental series used technical replicates (not biological replicates) as they examined acute effects. The number of such replicates is based on previous published observations to test statistical differences between similar datasets [as in del Villar S.G. et al, Proc Natl Acad Sci US A 118 (2021); Ito, D.W. et al, J Physiol 597, 2139-2162 (2019).; Dixon R.E. et al, Elife 4 (2015); Cheng E.P. et al, Circ Res 109, 255-261 (2011); Dixon R.E. et al, Proc Natl Acad Sci US A 109, 1749-1754 (2012)].
Data exclusions	Strict standards for isolation and cell quality were adhered to. Accordingly, cells were considered "healthy" and pursued for experiments and analysis when they fulfilled the following criteria:<br>Replication<br>(1) Cell isolation: at least 75 % cell survival was required to consider an isolation successful and to proceed with further evaluation and experiments.<br>(2) Cell morphology: "rod-shaped" /"brick-shaped" cells with clear striations, no blebbing, and no bunching at the cell ends.<br>(3) Cell stability: cells were required to be quiescent in physiological (1.8 mM Ca^{2+}-containing) solution, with no spontaneous action potentials and accompanying contractions.<br>(4) When electro physiology was performed, cells were pursued if they were found capable of holding stable seals with no more than 200 pA leak (but usually< 50 pA) for at least 10 mins.
Replication	To account for variability in sample preparation, animals, ambient conditions, etc, datasets are produced from cells from at least 3 different mice. All attempts at replication were successful.
Randomization	Treatments were acutely applied in cells that were randomly selected.
Blinding	Blinding was not possible for our experiments as they were performed before and after a specific acute treatment.

Reporting for specific materials, systems and methods

We require information from authors about some types of materials, experimental systems and methods used in many studies. Here, indicate whether each material, system or method listed is relevant to your study. If you are not sure if a list item applies to your research, read the appropriate section before selecting a response.

Materials & experimental systems

n/a	Involved in the study
☐	☒ Antibodies
☒	☐ Eukaryotic cell lines
☒	☐ Palaeontology and archaeology
☐	☒ Animals and other organisms
☒	☐ Clinical data
☒	☐ Dual use research of concern
☒	☐ Plants

Methods

n/a	Involved in the study
☒	☐ ChIP-seq
☒	☐ Flow cytometry
☒	☐ MRI-based neuroimaging

Antibodies

Antibodies used

rabbit polyclonal IgG anti-CaV1.2 (ACC-003, Alomone Labs; 1:300 for immunocytochemistry)

mouse monoclonal IgG1 anti-RyR2 (C3-33, MA3-916; 1:50 for immunocytochemistry)

mouse monoclonal IgG1 anti-EEA1 (ab70521, Abcam; 1:250 for immunocytochemistry)

mouse monoclonal IgG1 anti-Bin1 (2F11) (NBP2-21675, Novus Biologicals; 1:250 for western blots; 1:250 for immunocytochemistry)

mouse monoclonal IgG2b anti-Bin1 (99D) (sc-13575; Santa Cruz Biotechnology; 1:100 for western blots) or (05-449-C, Sigma-Aldrich; 1:500 for western blots)

mouse monoclonal IgG1 anti-cardiac troponin I (TI-4, AB_10573815; 1:100 for western blots; TI-4 was deposited by Schiaffino, S. to the Developmental Studies Hybridoma Bank, created by NICHD of the NIH and maintained at the University of Iowa, Department of Biology, Iowa City, IA 52242)

rabbit polyclonal anti-phospho-troponin I (Cardiac) (Ser23/24) (4004S, Cell Signaling Technology; 1:1000 for western blots)

anti-cMyBP-C 2-14 (kindly provided by Sakthivel Sadayappan, University of Cincinnati; 1:10,000 for western blots)

anti-phospho-cMyBP-C (Ser273) (kindly provided by Sakthivel Sadayappan, University of Cincinnati; 1:2500 for western blots).

Alexa Fluor 488 goat anti-rabbit (A-11034, Invitrogen; 1:1000)
Alexa Fluor 647 goat anti-mouse IgG1 (A-21240, Invitrogen; 1:1000)
Alexa Fluor 647 goat anti-rabbit (A-21245, Invitrogen; 1:1000)
Alexa Fluor 488 goat anti-mouse IgG1 (A-21121, Invitrogen; 1:1000)

Goat anti-mouse IgG (H+L)-HRP Conjugate (Biorad, 1706516; 1:10,000)
Monoclonal Mouse Anti-Rabbit IgG light chain specific HRP conjugate (Jackson ImmunoResearch, 211-032-171; 1:10,000)

Validation

All antibodies are commercially available or upon request by source, and the applications have been tested by the manufacturer and by us in different studies.

rabbit polyclonal IgG anti-CaV1.2 (ACC-003, Alomone Labs; 1:300)
validation: O. R. Buonarati, P. B. Henderson, G. G. Murphy, M. C. Horne, J. W. Hell, Proteolytic processing of the L-type Ca (2+) channel alpha 11.2 subunit in neurons. F1000Res 6, 1166 (2017). Data sheet: https://www.alomone.com/p/anti-cav1-2-antibody/ACC-003

mouse monoclonal IgG1 anti-RyR2 (C3-33, MA3-916; 1:50)
Data sheet: https://www.thermofisher.com/antibody/product/Ryanodine-Receptor-Antibody-clone-C3-33-Monoclonal/MA3-916

mouse monoclonal IgG1 anti-EEA1 (ab70521, Abcam; 1:250)
Citation: del Villar, S.G. PNAS 2021; Data Sheet: https://www.abcam.com/eea1-antibody-1g11-early-endosome-marker-ab70521.html

mouse monoclonal IgG1 anti-Bin1 (2F11) (NBP2-21675, Novus Biologicals; 1:250 for western blots; 1:250 for immunocytochemistry); anti-Bin1 (99D) (Santa Cruz Biotechnology; 1:100 for western blots) or (Sigma-Aldrich; 1:500 for western blots)
Validation: These antibodies were validated on BINI knockout tissues in the following publication: Targeted disruption of the murine Binl/Amphiphysin II gene does not disable endocytosis but results in embryonic cardiomyopathy with aberrant myofibril formation. Muller AJ, Baker JF, Du Hadaway JB, Ge K, Farmer G, Donover PS, Meade R, Reid C, Grzanna R, Roach AH, Shah N, Soler AP, Prendergast GC. Mal Cell Biol. 2003 Jun;23(12):4295-306. doi: 10.1128/MCB.23.12.4295-4306.2003. PMID: 12773571

mouse monoclonal IgG1 anti-cardiac troponin I (TI-4, AB_10573815; 1:100; TI-4 was deposited by Schiaffino, S. to the Developmental Studies Hybridoma Bank, created by NICHD of the NIH and maintained at the University of Iowa, Department of Biology, Iowa City, IA 52242)
Citation: Troponin I switching in the developing heart. Schiaffino S. The Journal of biological chemistry 264.27 (1989 Sep

25):16299-302

rabbit polyclonal anti-phospho-troponin I (Cardiac) (Ser23/24) (4004S, Cell Signaling Technology; 1:1000)
Data sheet: https://www.cellsignal.com/products/primary-antibodies/phospho-troponin-i-cardiac-ser23-24-antibody/4004?_requestid=7165805

anti-cMyBP-C 2-14 (kindly provided by Sakthivel Sadayappan, University of Cincinnati; 1:10,000 for western blots)
Citation: Sadayappan S, Gulick J, Osinska H, Barefield D, Cuello F, Avkiran M, et al. A critical function for ser-282 in cardiac Myosin binding protein-C phosphorylation and cardiac function. Circ Res. 2011;109(2):141-50.

anti-phospho-cMyBP-C (Ser273) (kindly provided by Sakthivel Sadayappan, University of Cincinnati; 1:2500 for western blots).
Citation: Sadayappan S, Gulick J, Osinska H, Barefield D, Cuello F, Avkiran M, et al. A critical function for ser-282 in cardiac Myosin binding protein-C phosphorylation and cardiac function. Circ Res. 2011;109(2):141-50.

Alexa Fluor 488 goat anti-rabbit (A-11034, Invitrogen; 1:1000)
Citation: del Villar, S.G. PNAS 2021
Datasheet: https://www.thermofisher.com/antibody/product/Goat-anti-Rabbit-IgG-H-L-Highly-Cross-Adsorbed-Secondary-Antibody-Polyclonal/A-11034

Alexa Fluor 647 goat anti-mouse IgG1 (A-21240, Invitrogen; 1:1000)
Citation: del Villar, S.G. PNAS 2021
Datasheet: https://www.thermofisher.com/antibody/product/Goat-anti-Mouse-IgG1-Cross-Adsorbed-Secondary-Antibody-Polyclonal/A-21240

Alexa Fluor 647 goat anti-rabbit (A-21245, Invitrogen; 1:1000)
Datasheet: https://www.thermofisher.com/antibody/product/Goat-anti-Rabbit-IgG-H-L-Highly-Cross-Adsorbed-Secondary-Antibody-Polyclonal/A-21245

Alexa Fluor 488 goat anti-mouse IgG1 (A-21121, Invitrogen; 1:1000)
Datasheet: https://www.thermofisher.com/antibody/product/Goat-anti-Mouse-IgG1-Cross-Adsorbed-Secondary-Antibody-Polyclonal/A-21121

Goat anti-mouse IgG (H+L)-HRP Conjugate (Biorad, 1706516; 1:10,000)
https://www.bio-rad.com/en-us/sku/1706516-goat-anti-mouse-igg-h-l-hrp-conjugate?ID=1706516

Monoclonal Mouse Anti-Rabbit IgG light chain specific HRP conjugate (Jackson ImmunoResearch, 211-032-171; 1:10,000)
https://www.jacksonimmuno.com/catalog/products/211-032-171

Animals and other research organisms

Policy information about studies involving animals; ARRIVE guidelines recommended for reporting animal research, and Sex and Gender in Research

Laboratory animals	University of California Davis Institutional Animal Care and Use Committee (IACUC) approved all procedures involving mice which were conducted in strict compliance with the Guide for the Care and Use of Laboratory Animals80 (protocol #s: 22848 and 21182). Mice were kept in a vivarium with a standard 12/12 light-dark cycle, a temperature range of 68 - 79 °F and humidity between 30 - 70 %. They received standard chow (Laboratory Rodent Diet 5001, LabDiet, St Louis, MO, USA) and water ad libitum. Mice were euthanized using an intraperitoneal injection of pentobarbital solution (> 100 mg/kg; B euthanasia-D Special; Merck & Co., Inc., Rahway, NJ, USA).<br><br>All experiments were performed on male mice. C57Bl/6 3-5-month-old (referred to as “young”) and 21-25-month-old mice (referred to as “old”) were sourced from the NIA Aged Rodent Colony (Charles River Laboratories) unless otherwise stated. In some experiments young C57Bl/6 mice sourced from The Jackson Laboratory (JAX; Sacramento, CA, USA) were utilized. Data from JAX-sourced young and NIA-sourced young mice are statistically compared in Supplementary Figure 1 and no significant differences were detected in any of the measured parameters. Figure legends specify which data were obtained from NIA-sourced or JAX-sourced young mice.
Wild animals	The study did not involve wild animals.
Reporting on sex	We clearly state in the title of the manuscript and in the methods that our experiments were performed on male mice.
Field-collected samples	The study did not involve any field-collected samples.
Ethics oversight	Animal studies were approved and overseen by the University of California Davis Animal Care and Use Committee (protocol #s: 22848 and 21182).

Note that full information on the approval of the study protocol must also be provided in the manuscript.

Plants

Seed stocks	N/A
Novel plant genotypes	N/A
Authentication	N/A
